# Analytical performance and clinical usability of a low-cost vaginal swab for sensitive detection of high-risk human papillomavirus

**DOI:** 10.12688/gatesopenres.16399.1

**Published:** 2026-10-15

**Authors:** Victor Omballa, Jared Luchetta, Rebecca Amaru, Vimarsh Joshi, Jessica Wicker, William Bennett, Leyla Solis, Bridgette Mastaglio, Ninette Cohen, Lindsey B Turnbull

**Affiliations:** 1 Quantigen, Fishers, Indiana, 46037, USA; 2 2. Brasner, Blumberg, Amaru and Olsen, LLP, New York, New York, 10128, USA; 3 Northwell New Hyde Park, Hempstead, New York, 11040, USA

**Keywords:** Human papillomavirus, low-cost, dry-stored, self-collected swabs

## Abstract

**Background:**

Cervical cancer remains a global health burden, with >600,000 new cases and 340,000 deaths reported in 2020. To reduce this burden, affordable and scalable human papillomavirus (HPV) diagnostics - ranging from at-home to centralized laboratory testing - are needed. Specimen collection plays a key role in analytical performance, sample integrity/stability, platform compatibility and user comfort.

**Methods:**

We evaluated the analytical performance and temperature stability of high-risk HPV (hrHPV) plasmid DNA and human cervical cancer cell lines on a low-cost Steripack XpressCollect flocked nylon swab using the Atila ScreenFire HPV risk stratification (RS) assay, the Cepheid Xpert HPV assay, and the Molbio Truenat HPV-HR Plus assay. We assessed the clinical usability among women seeking sexual and reproductive healthcare in New York, New York.

**Results:**

The Atila ScreenFire HPV RS assay was more sensitive in detection of HPV. We demonstrated statistically improved elution efficiency from the low-cost Steripack XpressCollect flocked nylon swab relative to Copan FLOQSwabs. Additionally, dry-stored flocked nylon swabs preserved sample integrity better than swabs stored in Tris-EDTA buffer over time with varying temperatures. Frozen HPV lysates remained stable for up to three months including during freeze/thaw cycles. Among healthcare provider-collected swabs, 7.5% were HPV positive and among the self-collected swabs, 20% were HPV positive. Of those with self-collected swabs, 97% had no previous self-swabbing experience, 71% preferred self-swabbing to healthcare provider-collected swabbing, 93% found it very easy or easy to self-swab and 100% were willing to provide self-swabbing at follow-up.

**Conclusion:**

The use of dry-stored, nylon-flocked vaginal swabs paired with platform-agnostic assays, like the Atila ScreenFire HPV RS and other genotyping assay, offers a practical and robust solution for hrHPV genotyping in resource-limited settings. Additionally, self-swabbing using low-cost vaginal swabs was highly accepted and retained equivalent sensitivity, further justifying its widespread use in underserved communities.

## Introduction

Cervical cancer incidence remains above World Health Organization (WHO) global standards and disproportionately affects those living in resource-limited settings where HPV screening programs are often insufficient to prevent progression of cancer due to a lack of options for early diagnosis and treatment. ^1^ Historically, cervical cancer screening has been performed using Papanicolaou (pap) smear cytology, which involved scraping cervical epithelial cells and examining them microscopically for abnormalities. ^2^ Pap smear cytology was the first screening method used to evaluate the risk and progression of cervical cancer in the 1960’s. ^2^ By the mid-1980’s, human papillomavirus (HPV) infection of cervical epithelial cells was identified as the main cause of cervical cancer. In the 1990’s, liquid based cytology (LBC) was introduced to address limitations of the pap smear including concerns for false negatives from reduced sample quality. ^3^ In the early 2000s, high-risk HPV (hrHPV) testing was incorporated into cervical cancer screening algorithms. ^4^ Currently, hrHPV testing is being implemented alongside cytological screening to improve accuracy in detecting high-grade lesions and to improve the overall diagnostic accuracy. ^5^ However, hrHPV testing alone has been recommended for primary screening in all settings since it can reduce the required amount of healthcare infrastructure, trained personnel, and specialized equipment. ^6^ hrHPV testing also has better scalability and is compatible with a “screen-and-treat” approach that limits loss to followup. ^6^ hrHPV testing, particularly when combined with self-swabbing, improves screening coverage, and outperforms cytology in identifying more people at risk of developing cervical cancer due to the tests’ ability to identify hrHPV genotypes that require monitoring. ^7^^–^^9^ Stratifying hrHPV genotypes using molecular testing may also help clinicians evaluate the risk of developing cervical cancer and prevent overtreatment, as HPV 16, 18, and 45 carry the highest risk of developing cervical cancer. ^10^^–^^12^

Sample collection for HPV screening has undergone a similar paradigm shift in the last decade including a global trend towards self-sampling. ^13^^,^^14^ Self-collection improves autonomy and privacy of sample collection, has the potential to expand where testing can occur at – home, in mobile clinics, or in healthcare settings – and offers comparable clinical performance. ^13^^,^^15^^,^^16^

Samples for cytology are stored in methanol-based media which is biologically hazardous and requires multiple complicated processing steps prior to molecular assay testing. Alcohol-free suspension media improves some of these logistical considerations, but dry stored swabs may offer an even better solution. ^16^^,^^17^ Studies, from our group and others, suggest that vaginal swabs for other sexually transmitted infections (STIs) remain stable when collected and stored dry for up to 30 days, requiring no refrigeration. ^16^^,^^18^^–^^20^ Self-collected vaginal swabs have been evaluated and found to be non-inferior to healthcare provider-collected cervical brush samples for hrHPV testing and highly acceptable for primary screening, thereby increasing HPV screening participation. ^14^^,^^21^^,^^22^ Self-collection and dry storage of swabs offer significant benefits for under screened populations because barriers to testing are removed, paving the way for decentralized hrHPV testing and the potential to reach underserved communities with high HPV disease burden. ^14^^,^^23^

In support of global access to HPV testing, the WHO recently established target product profile characteristics for point-of-care test developers, including a cost target of less than $5 per reportable result. ^24^ However, this benchmark is difficult to achieve when the cost of many self-collection devices is markedly high ranging from $41–50 per completed self-collected sample and varies by country, assay type and program design. ^25, 26^ In this study, we evaluate the analytical performance and elution efficiency of HPV DNA of HPV-related cell lines from Steripack XpressCollect flocked nylon swab (Steripack, Lakeland, FL, USA) compared to the widely used FLOQSwab flocked nylon swab (Copan Diagnostics, Murrieta, CA, USA). The Steripack XpressCollect flocked nylon swab was obtained for $0.10 to $0.30 per swab for this study. However, global pricing agreements might vary the cost. We further examine stability of HPV-related cell lines and lysate on the Steripack XpressCollect flocked nylon swab in different temperature storage conditions when stored dry or in buffer. Lastly, we describe HPV genotypes detected in healthcare provider-collected and self-collected vaginal swabs and clinical usability among women visiting a sexual and reproductive health clinic in New York, New York, USA.

## Methods

### Plasmid DNA amplification and clonal selection

Plasmid DNA containing whole genome HPV was obtained from the United States Centers for Disease Control and prevention (CDC, Atlanta, GA). ^27^ Briefly, HPV 16, HPV 18 and HPV 45 were amplified using NEB Stable Competent *Escherichia coli* (New England Biolabs, Ipswich, MA, USA) supplemented with 100 micrograms per milliliter (μg/mL) of carbenicillin antibiotics (Thermo Fisher Scientific, Waltham, MA, USA) in either LB broth or LB agar (Bio Basic, Markham, ON, Canada). *E. coli* was heat-shock transformed, spread onto LB agar selection plates, and incubated at 30°C for ~24 hours. Single colonies were picked and amplified in 2 mL of LB broth with selection antibiotics and then incubated at 30°C for ~24 hours. Plasmid DNA minipreps were performed using QIAprep Spin Miniprep Kit (Qiagen, Germantown, MD, USA), and clones were screened using restriction digests with PVuI-HF and BamHI-HF (New England Biolabs, Ipswich, MA, USA). Digested DNA was separated by gel electrophoresis and visualized to evaluate the presence of HPV positive colonies. These positive colonies were amplified in 100 mL LB broth with antibiotic selection at 30°C for ~24 hours. Plasmid DNA was purified using a HiSpeed Plasmid Midi Kit (Qiagen, Germantown, MD, USA) and quantified using a Nanodrop ND-1000 Spectrophotometer (Thermo Fisher Scientific, Wilmington, DE, USA). To minimize contamination risk, plasmids were diluted in Tris-EDTA (TE) buffer, pH 8.0 (Fisher Scientific, Waltham, MA, USA) to a final concentration between 1 and 10 ng/μL and stored at −20°C.

### Cell culture using human cervical cancer cell lines

Human cervical cancer cell lines including SiHa (ATCC HTB-35), HeLa (ATCC CCL-2), CaSki (ATCC CRL-1550), and C-33A (ATCC HTB-31) cells from American Type Culture Collection (ATCC, Manassas, VA, USA) were grown at 37°C with 5% CO _2_ in culture media: Dulbecco’s Modified Eagle Medium high glucose with sodium pyruvate containing 10% Fetal Bovine Serum (Gibco, Thermo Fisher Scientific, Waltham, MA, USA) and 50 μg/mL gentamycin (Sigma-Aldrich, St. Louis, MO, USA) under standard aseptic conditions while screening for mycoplasma contamination using the Universal Mycoplasma Detection kit (ATCC, Manassas, VA, USA) according to the manufacturer’s instructions.

### Contriving flocked nylon swabs

Either the Steripack XpressCollect flocked nylon swabs or Copan FLOQSwabs were contrived using different concentrations of plasmid DNA or human cervical cancer cell lines (shown above).

For swabs contrived using DNA, the plasmids were serially diluted in nuclease-free water to the required copy number per swab, then mixed with 100 ng of HPV-negative C-33A genomic DNA. A final volume of 15 μL of plasmid DNA was prepared and transferred to 2 mL microcentrifuge tubes (Invitrogen, Thermo Fisher Scientific, Carlsbad, CA, USA) prior to lysis. Swabs contrived with human cervical cancer cell lines were prepared during sub-culture by resuspending and serially diluting cells in Dulbecco’s Phosphate-Buffered Saline (DPBS), Gibco (Thermo Fisher Scientific, Waltham, MA, USA) and counted using a hemocytometer. A 15 μL aliquot containing the desired number of human cervical cancer cell line was transferred to individual 2 mL microcentrifuge tubes prior to lysis.

For dry-stored swabs, a sterile swab was inserted into the 2 mL microcentrifuge tubes, rotated to ensure complete absorption of the 15 μL suspension (mentioned above) and subsequently broken at the 30 mm breakpoint. For buffered swabs, following complete absorption of the 15 μL suspension, 150 μL of Tris-EDTA (TE) buffer, pH 8.0 was added prior to lysis.

### Isothermal amplification using Atila ScreenFire HPV RS assay

We utilized the Atila ScreenFire HPV RS assay (Atila BioSystems, Sunnyvale, CA, USA) isothermal amplification protocol to test swab samples on QuantStudio 5/5Dx real-time PCR instrument. This assay detects 13 hrHPV genotypes grouped into four risk-based categories including group 1 (HPV 16), group 2 (HPV 18/45), group 3 (HPV 31/33/35/52/58) and group 4 (HPV 39/51/56/59/68) in four separate fluorescent detection channels. ^28^ Briefly, contrived swabs in 2 mL microcentrifuge tubes were lysed using 1 mL of 1X lysis buffer (Atila BioSystems, Sunnyvale, CA, USA) and then vortexed for 10–15 seconds before incubation at 95°C for 20 minutes. After incubation, swab samples were allowed to cool to room temperature (~20°C) before proceeding to isothermal amplification.

The reaction master mix for the Atila ScreenFire HPV RS assay was prepared using, 10 μL of reaction mix (HPVM4FRM), 10 μL of primer mix (HPVM45PM), and 5 μL of lysate in each well of the MicroAmp Optical 96-well reaction plates with barcodes (Applied Biosystems, Thermo Fisher Scientific, Carlsbad, CA, USA), yielding a total reaction volume of 25 μL. Plates were vortexed for 30 seconds, centrifuged prior to loading on the instrument. Isothermal amplification was completed over 60 cycles, each consisting of a one-minute incubation at 60°C, with fluorescence measurements acquired at the end of each cycle. Data were analyzed using QuantStudio 5 Software v1.2.0, with baseline fluorescence calculated between cycles 3 and 15 using the following thresholds expressed as dye, HPV target, and threshold setting: CY5, HPV 16, 40,000; ROX, HPV 18/45, 30,000; CY5.5, HPV 31/33/35/52/58, 20,000; FAM, HPV 39/51/56/59/68, 30,000; HEX, Internal Control (human β-globin); 10,000. For further HPV genotyping, the reaction master mix for the Atila AmpFire HPV high-risk genotyping assay (Atila BioSystems, Sunnyvale, CA, USA) was performed according to the manufacturer’s instructions. ^29^

This isothermal amplification assay qualitatively detects 15 hrHPV (i.e., HPV 16, 18, 31, 33, 35, 39, 45, 51, 52, 53, 56, 58, 59, 66 and 68) using several dyes including CY5 (HPV 16), ROX (HPV 18), FAM (all other HPV genotypes) and HEX for internal control (human β-globin) as shown above. ^30^

### Limit of Detection (LoD) using contrived Steripack XpressCollect flocked nylon swabs

*LoD using plasmid DNA:* Whole genome HPV 16, 18, or 45 plasmid DNA from the US CDC were serially diluted 1:2 in 2 mL microcentrifuge tubes using nuclease free water to final concentrations ranging from 12,500 copies/mL to 12 copies/mL. Each swab received HPV plasmid DNA and 100 ng purified C-33A genomic DNA in a total volume of 50 μL. Swabs were either eluted with Atila 1X lysis buffer specific for the Atila ScreenFire HPV RS assay or with nuclease free water for the Cepheid GeneXpert HPV assay (Cepheid, Sunnyvale, CA, USA) and Molbio Truenat HPV-HR assay (Molbio Diagnostics, Goa, India). For isothermal amplification platform comparison, only HPV 16 plasmid DNA was used with replicated experiments occurring over three different days. Lysate input volumes for each of the platforms were added according to each platform’s Instructions for use i.e., 5 μL for the Atila ScreenFire HPV RS assay, 1 mL for Cepheid GeneXpert HPV assay, 6 μL for Molbio Truenat HPV-HR assay. Data was generated using the Thermo Fisher QuantStudio 5/5Dx, Cepheid GeneXpert Instrument System, or Molbio Truelab Real Time Quantitative micro–PCR Analyzer.

*LoD using human cervical cancer cell lines:* SiHa (ATCC HTB-35), HeLa (ATCC CCL-2), and CaSki (ATCC CRL-1550) cells, were serially diluted 1:2 in 2 mL microcentrifuge tubes to final concentrations ranging from 400,000 cells/mL to 6 cells/mL. Samples were contrived onto the swabs as previously outlined.

### Elution efficiency of HPV from two different flocked nylon swab types

We evaluated the elution efficiency of HPV DNA or HPV-related cell lines from two different flocked nylon swabs i.e., Steripack XpressCollect and Copan FLOQSwab using HPV 16 plasmid DNA or SiHa cells only.

The flocked nylon swabs were contrived at 2X and 10X of the LoD and testing was done using the Atila ScreenFire HPV RS assay. Eight swabs per day were contrived and the experiment was replicated over 3 days, resulting in a total of 24 swabs per HPV 16 genotype group.

### HPV stability on dry or buffered Steripack XpressCollect flocked nylon swabs

*Constant Temperature Evaluations:* To assess the feasibility of using the Steripack XpressCollect flocked nylon swabs in settings where refrigeration may be limited or unavailable, and to evaluate whether the non-toxic Tris-EDTA buffer could serve as a suitable transport and storage medium following healthcare provider- or self-collected swabs, we contrived low-cost swab with 2X LoD of either HPV 16 plasmid DNA, SiHa cells (ATCC HTB-35), or HeLa cells (ATCC CCL-2) with no matrix, and stored swabs at constant temperatures of either 20°C or 40°C until isothermal amplification at specific time points including baseline (0 hours), 24 hours, 72 hours, 7 days, 14 days, and 30 days. We utilized 20 replicates per time point for each temperature.

*Evaluation of Temperatures simulating Transport conditions:* To assess whether swabs stored dry remain stable during temperature fluctuations like those expected during summer transport conditions, we conducted a follow-up study using only Steripack XpressCollect flocked nylon swabs. Up to 20 dry-stored flocked nylon swabs per time point were contrived with either 2X LoD SiHa or HeLa cells and then subjected to temperature fluctuations between 40°C, 30°C and 20°C across 104 hours to mimic transportation of samples from the collection site to a centralized laboratory for testing during summer months. These swabs were tested using Atila ScreenFire HPV RS assay at 24-hour increments plus an additional 8-hour delay to simulate a scenario in which swabs arrive but are not tested until the end of a work shift.

Temperatures and processing timepoints were chosen based on recommendations in the U.S. FDA’s Template for Developers of Home Specimen Collection Devices for Use with Molecular Diagnostic Tests. ^29^ Samples were processed following the Atila ScreenFire HPV RS assay methods outlined above.

*HPV lysate stability:* Following lysis using 1 mL of Atila 1X lysis buffer, up to 20 HPV lysates each from SiHa and HeLa cells previously contrived on swabs stored dry and then subjected to either a consistent −20°C storage or up to six freezer/thaw cycles to evaluate long-term HPV lysate stability. The HPV lysates were tested using the Atila ScreenFire HPV RS assay at six measured time points including baseline (0 hours), 48 hours, 7 days, 30 days, 2 months, and 3 months.

### Clinical collections and usability of Steripack XpressCollect flocked nylon swab

Women seeking general gynecological exams and regular hrHPV screening from a general obstetrics and gynecological practice in New York, NY, USA were given a short description of the study following a written informed consent. Consenting study participants were assigned a unique study identification number for de-identification purposes and asked to complete a novel and structured questionnaire about demographic information. For Phase 1 of the SHIELDS (Sensitive hrHPV Infection Early Low-cost Detection using Self-collected swabs) study that used convenience sampling, healthcare providers collected up to five study-related vaginal swabs prior to patient-related standard of care procedures and self-reporting of vaccination status. For Phase 2 of the SHIELDS study using convenience sampling, women self-collected up to three vaginal swabs following instructions for use. These swabs were then shipped to Quantigen for HPV genotyping using the Atila ScreenFire HPV RS assay and the AmpFire HPV high-risk genotyping assay (Atila BioSystems, Sunnyvale, CA, USA). For the women who self-collected swabs, we evaluated usability of the Steripack XpressCollect flocked nylon swab to understand the acceptability and user experience, ease of sampling, pain/discomfort, confidence in self-sampling and trust in results, existing concerns or worries and their willingness to use or recommend self-swabbing.

### Statistical considerations

Data was exported to Microsoft Excel 365 (Microsoft Corporation, Redmond, WA, USA) and GraphPad Prism 10 (GraphPad Software, Boston, MA, USA) for further statistical analysis and graphical representation. Probit analysis was used to estimate the LoD of the Atila ScreenFire HPV RS assay compared to the Cepheid GeneXpert HPV assay and the Molbio Truenat HPV-HR assay. The estimated LoD was defined as the concentration at which 95% HPV positivity is detected from the Steripack XpressCollect flocked nylon swab. The mean and standard deviation of the cycle threshold (Ct) and one-way analysis of variance (ANOVA) were used to compare elution efficiency of HPV from the different flocked nylon swabs compared to plasmid DNA or human cervical cancer cells with p-value less than 0.05 considered statistically significant. Categories of hrHPV genotypes and swab usability data are presented as frequencies and percentages.

## Results

### Analytical sensitivity using HPV 16 plasmid DNA on low-cost flocked nylon swab

Using the Steripack XpressCollect flocked nylon swab spiked with HPV 16 whole genome, the interpolated LoDs at 95% positivity were 1479 (3.16 log) copies/mL, 5248 (3.72 log) copies/mL, and 208,930 (5.32 log) copies/mL for the Atila ScreenFire HPV RS assay, the Cepheid GeneXpert HPV assay, and the Molbio Truenat HPV-HR assay respectively ( Figure 1). The Atila ScreenFire HPV RS assay has similar LoD to the Cepheid GeneXpert HPV assay (i.e., 3.16 vs 3.72 log copies/mL) while the Molbio Truenat HPV-HR assay demonstrated a higher LoD.

**Figure 1.**
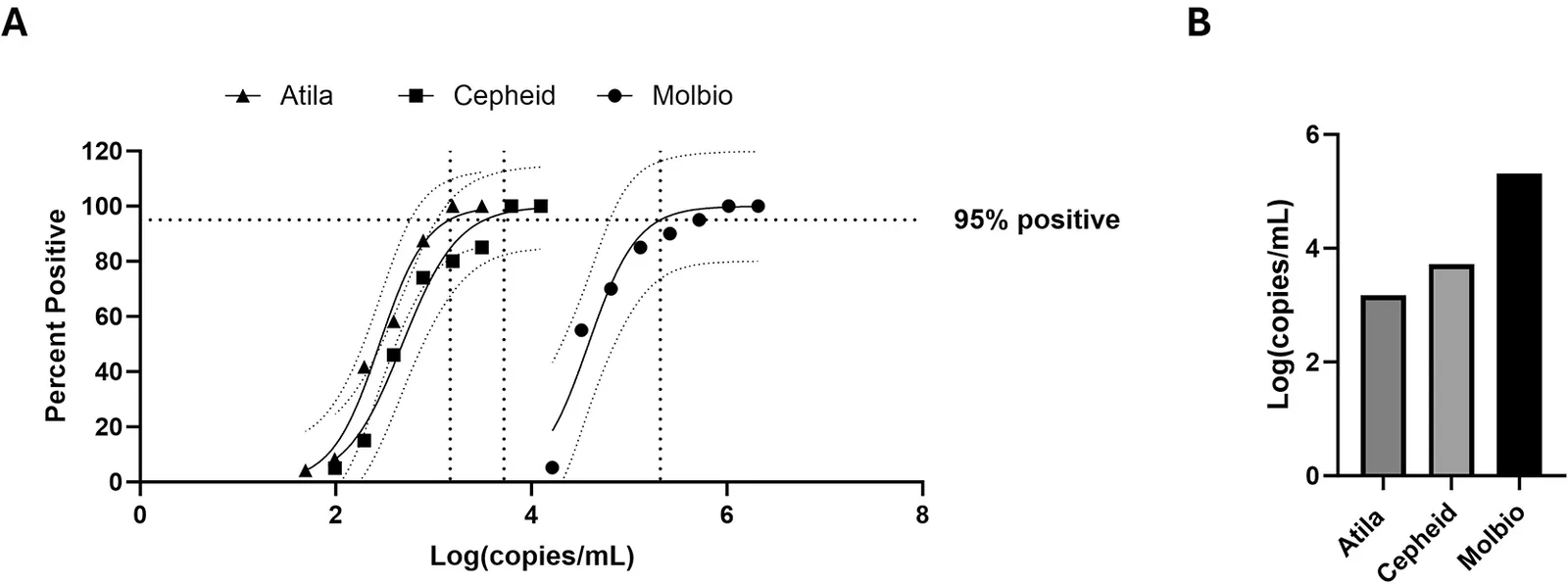
Logarithmic curves for the Atila ScreenFire HPV RS assay (n = 24), Cepheid GeneXpert HPV assay (n = 20), and Molbio Truenat HPV-HR assay (n = 20). HPV assays LoDs were calculated based on standard curve concentration to determine percent positivity at various concentrations of HPV 16 plasmid DNA expressed in copies per mL (A). Interpolated concentration at 95% sample positivity for each HPV test platform (B).

We further determined the LoD of the Steripack XpressCollect flocked nylon swab paired with the Atila ScreenFire HPV RS assay for additional HPV genotypes including whole genome HPV 16, and18; HPV 45 plasmid DNA; and human cervical cancer cell lines including SiHa, CaSki, and HeLa cells. The estimated LoD for whole genome HPV 16, whole genome HPV 18, and HPV 45 plasmid DNA was 1600 copies per mL, 800 copies per mL, and 800 copies per mL respectively ( Table 1). The LoD for SiHa, CaSki, and HeLa cells was 400 cells per mL, 6 cells per mL and 200 cells per mL respectively ( Table 1).

**Table 1.** Limit of Detection of different HPV genotypes on the Steripack XpressCollect flocked nylon swab on the Atila ScreenFire HPV RS assay.

HPV genotype	Plasmid DNA LoD (copies/mL)	Human cervical cancer cell lines LoD (cells/mL)
HPV 16	1600	400 (SiHa)
		6 (CaSki)
HPV 18	800	200 (HeLa)
HPV 45	800	N/A

### Elution efficiency of HPV DNA or HPV-related cell lines from two different XpressCollect flocked nylon swab types

When testing for elution efficiency using HPV 16 plasmid DNA at 2X LoD, the mean Ct values were similar between the HPV 16 plasmid no swab control group (27.71, SD ±4.73) and the Steripack XpressCollect flocked nylon group (27.24 SD ± 2.66) with no statistically significant difference (p = 0.9659), indicating high elution efficiency from the Steripack XpressCollect flocked nylon swab ( Figure 2A). In contrast, the Copan FLOQSwab group had significantly elevated Ct values (42.08, SD ± 9.76, p < 0.0001), with 4/20 (20%) samples failing to amplify, resulting in a percent positivity of 80%. Additionally, the Steripack XpressCollect flocked nylon swabs eluted HPV 16 plasmid DNA more efficiently than the Copan FLOQSwab (p < 0.0001).

**Figure 2.**
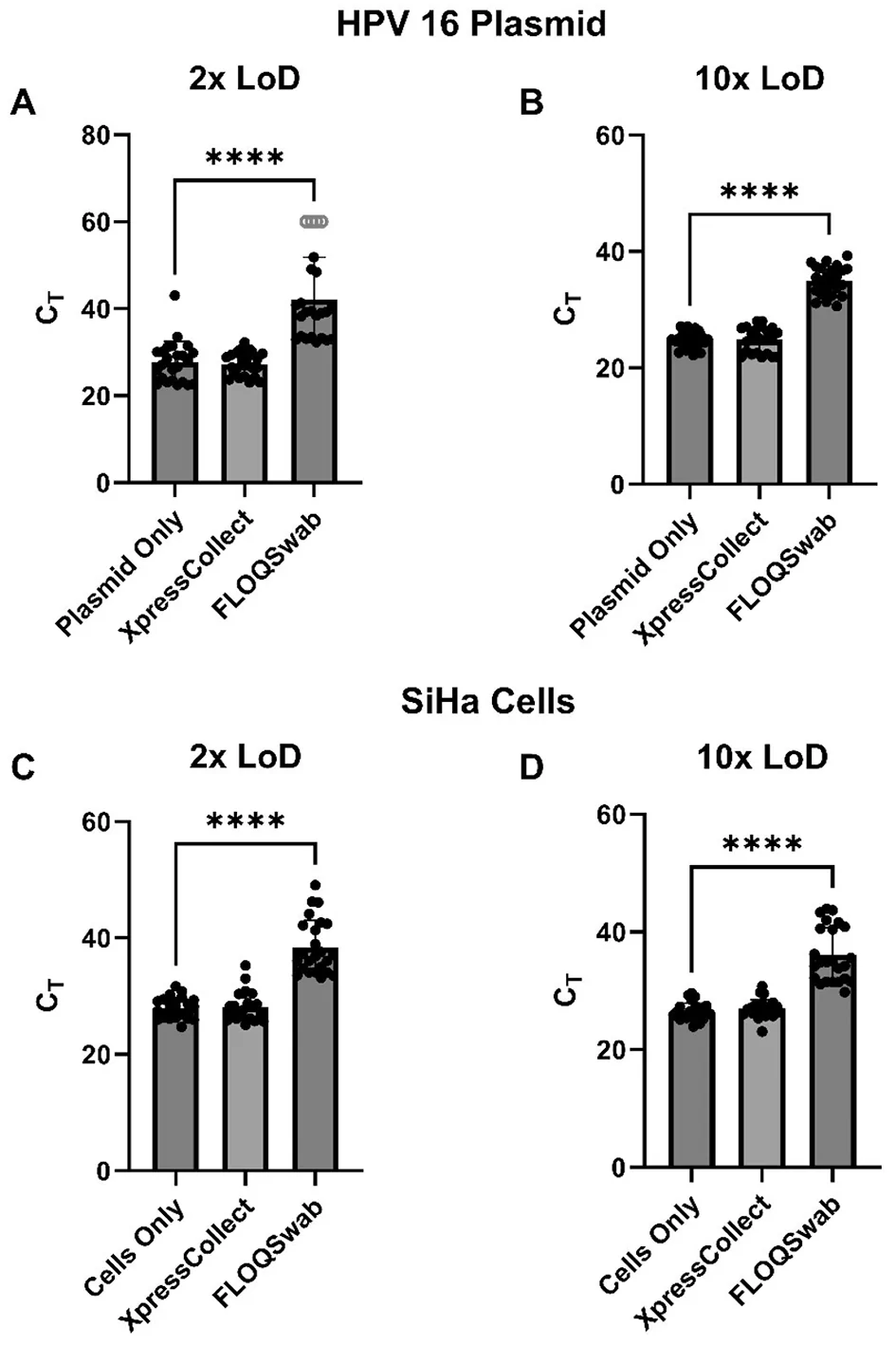
Elution efficiency of HPV DNA and HPV-related cell lines from two different flocked nylon swabs. Ct values of Steripack XpressCollect flocked nylon swabs and Copan FLOQSwabs contrived with HPV 16 plasmid DNA at 2X LoD (A) and 10X LoD (B) tested using the Atila ScreenFire HPV RS assay. Ct values of Steripack XpressCollect flocked nylon swabs and Copan FLOQSwabs contrived with SiHa cells at 2X LoD (C) and 10X LoD (D) using the Atila ScreenFire HPV RS assay. One-way ANOVA ****p < 0.0001.

At 10X LoD, similar trends in mean Ct values were observed for the HPV 16 plasmid no swab control group (24.80, SD ± 1.44), Steripack XpressCollect flocked nylon (24.85, SD ± 2.13), and Copan FLOQSwabs (35.00, SD ± 2.49) ( Figure 2B). There was no statistically significant difference between the HPV 16 plasmid no swab control group at 10X LoD and the Steripack XpressCollect flocked nylon swab (p = 0.9961), while a significant difference was noted between both the HPV 16 plasmid no swab control group at 10X LoD and the Steripack XpressCollect flocked nylon swab group relative to the Copan FLOQSwab (p < 0.0001 for both). However, all samples in the Copan FLOQSwab group amplified indicating that when using Copan FLOQSwabs this concentration was above the previously determined assay LoD.

For Swabs contrived with SiHa cells at 2X LoD, the mean Ct values for cell-only control (27.95, SD ± 1.77), Steripack XpressCollect flocked nylon swabs (28.19, SD ± 2.51), and Copan FLOQSwabs (38.43, SD ± 4.67) ( Figure 2C) demonstrated higher elution efficiency of cells from the Steripack XpressCollect flocked nylon swabs, with Copan FLOQSwabs yielding significantly higher Ct values relative to either the SiHa cell-only group or the Steripack XpressCollect flocked nylon swab group (p < 0.0001 for both). At 10X LoD, the trend in Ct values persisted for cell-only control (26.44, SD ± 1.46), Steripack XpressCollect flocked nylon swabs (26.97, SD ± 1.55), and Copan FLOQSwabs (36.11, SD ± 4.64) ( Figure 2D).

We observed a statistically significant difference between the Copan FLOQSwabs relative to the no swab control groups (i.e., HPV 16 plasmid DNA or SiHa cells only), p < 0.0001 at both the 2X LoD and 10X LoD concentrations. Similarly, there was statistically significant higher elution efficiency from the Steripack XpressCollect flocked nylon swab relative to the Copan FLOQSwabs group at different concentrations (p < 0.0001). There was no statistically significant difference when comparing Steripack XpressCollect flocked nylon swabs to either the HPV 16 plasmid DNA at 2X LoD or at 10X LoD (i.e., p = 0.9659 and p = 0.9961 respectively) or the SiHa cells control at 2X or at 10X (i.e., p = 0.9636 and p = 0.8041 respectively) highlighting the higher elution efficiency of HPV DNA and HPV-related cell lines from the Steripack XpressCollect flocked nylon swab ( Figure 2).

### HPV genetic material stability using dry or buffered universal Steripack XpressCollect flocked nylon swabs

Swabs contrived with HPV 16 plasmid DNA, SiHa cells (HPV 16), and HeLa cells (HPV 18) and stored dry remain stable at both 20°C (room temperature) and 40°C across all time points for up to 30 days ( Table 2). Conversely, swabs stored in Tris-EDTA buffer contrived similarly and tested under the same temperature conditions demonstrated variable stability depending on the source HPV genetic material and storage temperature. At 20°C, Tris-EDTA buffered swabs contrived with HPV 16 plasmid DNA were stable for up to 14 days, those contrived with SiHa cells remained stable for up to 30 days, while those contrived with HeLa cells were only stable for only 24 hours. At 40°C, Tris-EDTA buffered swabs remained stable for 72 hours with HPV 16 plasmid DNA, only at baseline for SiHa cells, and up to 30 days with HeLa cells ( Table 2).

**Table 2.** Comparison of HPV stability on dry or Tris-EDTA buffered Steripack XpressCollect flocked nylon swabs at different temperatures and time points.

Dry flocked nylon swab stability							
Temperature	Genetic material	T _0_	24 hr	72 hr	7d	14d	30d
20°C	HPV16 plasmid	20/20	20/20	20/20	20/20	19/20	**20/20**
	SiHa cells (HPV 16)	20/20	20/20	20/20	20/20	20/20	**20/20**
	HeLa cells (HPV 18)	20/20	20/20	20/20	19/20	20/20	**20/20**
40°C	HPV16 plasmid	20/20	20/20	20/20	20/20	19/20	**19/20**
	SiHa cells (HPV 16)	20/20	20/20	20/20	20/20	20/20	**20/20**
	HeLa cells (HPV 18)	19/20	20/20	20/20	20/20	19/20	**20/20**

Tris-EDTA buffered flocked nylon swab stability							
Temperature	Genetic material	T _0_	24 hr	72 hr	7d	14d	30d
20°C	HPV16 plasmid	20/20	20/20	20/20	20/20	**20/20**	18/20
	SiHa cells (HPV 16)	20/20	19/20	20/20	20/20	20/20	**20/20**
	HeLa cells (HPV 18)	20/20	20/20	18/20	19/20	19/20	15/20
40°C	HPV16 plasmid	20/20	20/20	**20/20**	18/20	15/20	16/20
	SiHa cells (HPV 16)	**20/20**	16/20	4/20	2/20	0/20	1/20
	HeLa cells (HPV 18)	20/20	20/20	20/20	20/20	20/20	**20/20**

Dry-stored flocked nylon swabs remained stable throughout the 104 hours for both SiHa or HeLa cells ( Table 3), with no significant loss of HPV stability observed at any of the 8-hour time points (highlighted in gray), including the extended 96 + 8 hr. condition.

**Table 3.** Comparison of SiHa (HPV 16) and Hela (HPV 18) cells stability on dry-stored Steripack XpressCollect flocked nylon swabs with fluctuating temperatures.

Day	Time	Temperature	Cycle period	Total time hours	SiHa	HeLa
1	7:00 AM	0 h			20/20	20/20
	7:00 AM	40°C	1	0		
	3:00 PM	20°C	2	8		
	7:00 PM	40°C	3	12		
	9:00 PM	30°C	4	14		
2	3:00 PM	24 + 8 h (32 h)			20/20	20/20
3	9:00 AM	40°C	5	50		
	3:00 PM	48 + 8 h (56 h)			20/20	20/20
	3:00 PM	20°C	6	56		
	7:00 PM	40°C	7	60		
	9:00 PM	30°C	8	62		
4	3:00 PM	72 + 8 h (80 h)			20/20	20/20
5	9:00 AM	40°C	9	98		
	3:00 PM	96 + 8 h (104 h)			20/20	20/20

Multiple replicates of HPV lysates stored at −20°C remained stable across all six measured time points with consistent HPV detection in 20 replicates per time point for both human cervical cancer cell lines, indicating no loss of HPV lysate stability over the full storage period ( Table 4). When subjected to six freeze/thaw cycles, HPV lysates from SiHa cells showed a reduction in stability with <95% HPV positivity after three freeze thaws at the 30-day time point. HPV lysates from HeLa cells retained stability across all testing time points despite freeze/thaw cycles. Overall, lysates derived from HPV-related cell lines and tested using the Atila ScreenFire HPV RS assay exhibited excellent long-term stability up to three months when stored at −20°C, though repeated freeze/thaw cycles reduced HPV lysate stability in a sample-dependent manner ( Table 4).

**Table 4.** SiHa (HPV 16) and Hela (HPV 18) cells lysate stability when frozen compared to multiple freezer/thaw cycles.

A								
Lysate Stability (−20°C)								
Sample	Condition	Swab	T _0_	48 hr	7d	30d	2mo	3mo
**SiHa (HPV 16)**	**Constant − 20°C**	**Dry**	20/20	20/20	19/20	19/20	19/20	**20/20**
**HeLa (HPV 18)**	**Constant − 20°C**	**Dry**	20/20	20/20	20/20	20/20	20/20	**20/20**

B								
Lysate Stability (Freeze/Thaw)								
Sample	Condition	Swab	T _0_	48 hr 1 F/T *	7d 2 F/T *	30d 3 F/T *	2mo 4 F/T *	3mo 5 F/T *
**SiHa (HPV 16)**	**Freeze/Thaw**	**Dry**	20/20	20/20	**20/20**	18/20	19/20	19/20
**HeLa (HPV 18)**	**Freeze/Thaw**	**Dry**	20/20	20/20	20/20	20/20	20/20	20/20

* F/T, Freeze thaw.

### HPV genotyping of clinically collected universal Steripack XpressCollect flocked nylon swabs and usability

In Phase 1 of the SHIELDS study, we tested 53 healthcare provider-collected vaginal swabs using the Atila ScreenFire HPV RS assay and completed individual hrHPV genotyping using the Atila AmpFire HPV high-risk genotyping assay. Of these, 4/53 (7.5%) were positive for HPV Group 4 of the assay. On further individual genotyping using the AmpFire HPV high-risk genotyping assay, the specific genotypes for these four study participants were HPV 39, HPV 51, HPV 56 and HPV 68. Self-reported HPV vaccination rate was moderately low among those with an HPV test result i.e., 47% (23/49) and most of the study participants were Caucasian 35/52 (67%) with an average age of 35 years.

In Phase 2 of the SHIELDS study, we evaluated the usability of the Steripack XpressCollect flocked nylon swab among a separate group of 40 women who provided self-collected vaginal swabs ( Table 5). We had varying responses to factors being evaluated demonstrated by the differences in the denominator ( Table 5). Results were therefore calculated using the number of completed observations for each factor of interest. Most women had no previous self-swabbing experience (97%) but were willing to provide self-swabbing again (100%).

**Table 5.** Factors associated with usability of self-collected Steripack XpressCollect flocked nylon swabs.

Domain	Measure	Responding (n/N)	Results
Acceptability and experience	Reported not having any previous self-swabbing experience	35/36	97%
	Reported preference for self-swabbing to healthcare-provider swabbing at clinic visit	27/38	71%
	Reported that self-swabbing instruction were clear	36/38	94.70%
	Reported no challenges when self-swabbing	34/39	87.2%
	Reported “excellent or good” self-swabbing experience at the clinic visit	38/40	95%
Ease of self-swabbing	Reported self-swabbing procedure as “Very easy/easy”	37/40	92.5%
	Reported “standing without lifting a leg or lifting one leg” during self-swabbing	32/40	80%
Pain and discomfort	Reported “very comfortable or somewhat comfortable” with self-swabbing	38/40	95%
	Reported “No” discomfort during self-collection	26/40	65%
Confidence in self-swabbing and trust in results	Reported “very confident or somewhat confident” with self-swabbing	35/40	87.5%
Concerns and worries	Reported no concerns about hygiene and safety of self-swabbing	39/40	97.5%
	Reported self-swabbing feeling more private	40/40	100%
Willingness to use/recommend self-swabbing	Reported willingness to provide self-swabbing again	39/39	100%
	Reported not being anxious or embarrassed about self-swabbing	39/40	97.5%
	Strongly agree or agree to recommend self-swabbing to others	34/40	85%

On hrHPV genotyping using the Atila ScreenFire HPV RS assay, 5/40 (12.5%) were positive for HPV Group 4 only. In addition, we detected alpha-9 HPV genotype (HPV Group 3) in 2/40 (5.0%) self-collected vaginal swabs and one participant (1/40; 2.5%) was positive for HPV 16 genotype and was coinfected with HPV Group 4; this co-infected case was not included in the HPV Group 4–only total.

## Discussion

Analytical sensitivity in HPV detection depends on numerous factors including method of sample collection, swab type used, the assay, and the platform used to test. We demonstrated improved HPV detection of the HPV 16 genotype across different testing platforms following direct lysis using manufacturer specific lysis workflow including Atila ScreenFire HPV RS assay, Cepheid GeneXpert HPV assay, and MolBio Trunat assay paired with the Steripack XpressCollect flocked nylon swab. The Atila ScreenFire HPV RS assay demonstrated the best analytical sensitivity. We utilized this assay and demonstrated an acceptable LoD for HPV-related plasmid DNA and HPV cell lines including HPV 16 (1479 copies/ml), HPV 18 (800 copies/mL), HPV 45 (800 copies/mL), SiHa (400 cells), CaSki (6 cells) and HeLa (200 cells).

We evaluated three hrHPV testing platforms that have potential for broad use at the point of care. The Atila ScreenFire HPV RS assay and Cepheid GeneXpert HPV assay had similar LoD; however, the Atila ScreenFire HPV RS assay has the additional advantage of being compatible with multiple plate-based real-time fluorescent detection instruments and a higher throughput. As such, it is a more adaptable assay enabling broader global deployment while the Cepheid GeneExpert HPV assay requires specific equipment that might not be as accessible with limited flexibility.

Elution efficiency of HPV DNA and HPV-related cell lines was evaluated for the Steripack XpressCollect flocked nylon swab relative to the widely used Copan FLOQSwab with the objective of providing more options for sample collection devices including swabs that could lower the cost of HPV diagnostic testing. The low-cost Steripack XpressCollect flocked nylon swab allowed HPV to be almost completely eluted from the swab material for both plasmid and cell lines at different concentrations. This elution efficiency off the swab was demonstrated by quantitative PCR in which the eluted material amplified within the same cycle as spiked buffer without a swab. However, there was significantly lower elution efficiency of HPV-related cell lines and DNA from the Copan FLOQSwab.

When testing using the Atila ScreenFire HPV RS assay, the Copan FLOQSwab failed to consistently elute sufficient HPV, particularly near the assay’s LoD, resulting in reduced HPV detection rates below the 95% positivity threshold. These findings are consistent with our previous work showing that more aggressive lysis methods, such as vortex bead beating, improves elution from Copan FLOQSwabs. ^18^ It is likely that the standard lysis conditions used by the Atila ScreenFire HPV RS assay are insufficient for complete HPV recovery from Copan FLOQswabs demonstrated by significantly higher Ct values. Incorporating a more vigorous agitation step may enhance elution efficiency especially when using the Copan FLOQSwabs. We did not evaluate swab capture efficiency relative to release of HPV DNA for detection. However, taken together, these results support the use of the Steripack XpressCollect flocked nylon swab, due to its more reliable elution efficiency of HPV DNA under standard processing conditions and possibly better suitability for simplified workflows.

Swab elution efficiency can be influenced by factors such as the swab’s chemical composition, flocking density, fiber length, and material type. Previously, flocked nylon swabs have been shown to have high clinical sensitivity when paired with downstream applications. ^15^ However, it is important to note that within different flocked nylon swabs, subtle differences exist that influence the efficacy of HPV elution efficiency as demonstrated by our studies and others ^15^^,^^31^ in which the Steripack XpressCollect and Copan FLOQSwab had markedly different analytical elution efficiencies.

HPV sample stability is a critical consideration across laboratory diagnostic workflows, as it directly impacts test accuracy and reliability regardless of setting. Many clinics in low-resource settings do not have ample and consistent access to cold storage, so evaluation of sample stability across various ambient temperatures and transportation conditions is important for increased screening efforts.

We assessed the stability of HPV on contrived Steripack XpressCollect flocked nylon swabs under different storage conditions using HPV 16 plasmid DNA, SiHA cells (HPV 16) and HeLa cells (HPV 18) since these HPV genotypes are strongly associated with development of cervical cancer and are of the most global importance. ^32^

Firstly, we compared Steripack XpressCollect flocked nylon swabs stored dry to those stored in Tris-EDTA buffer at constant temperatures of either 20°C or 40°C. Regardless of storage temperature, dry-stored flocked nylon swabs consistently preserved HPV genetic material integrity up to 30 days. We hypothesize that this could be due to reduced hydrolysis, oxidation, and alkylation. However, HPV on Steripack XpressCollect flocked nylon swabs suspended in Tris-EDTA buffer demonstrated variability in stability over time. When we subjected the dry-stored Steripack XpressCollect flocked nylon swabs containing either SiHa (HPV 16) and HeLa (HPV 18) cells to fluctuating temperatures, the swabs remained stable and reliably detectable—even near the assay’s limit of detection. We further subjected the lysates from the SiHa (HPV 16) and HeLa (HPV 18) cells to either constant storage at −20°C or six freeze/thaw cycles.

HPV lysate remained stable and reliably detected when stored frozen. However, with increased freeze thaw cycles, we observed instability in SiHa cell (HPV 16) samples. Altogether, these results highlight the robustness of flocked nylon swabs stored dry and support their use in laboratory workflows where maintaining sample integrity is essential, including during shipping, field collection, or decentralized testing. In addition, flocked nylon swabs stored dry are cost effective, eliminating the need for cold chain and have reduced biosafety requirements. Dry-stored swabs reduce logistical challenges experienced when using toxic or non-toxic buffered swabs which are more costly. ^16^^,^^31^

Following our laboratory workflow optimization, we tested healthcare provider-collected and self-collected vaginal swabs using the Steripack XpressCollect flocked nylon swab and highly sensitive Atila ScreenFire HPV RS assay. We detected some hrHPV genotypes associated with precancerous lesions.

We also demonstrated that the Steripack XpressCollect flocked nylon swab is highly acceptable among women despite not having self-swabbed prior to the study. We demonstrated that our user instructions were easy to understand and did not pose challenges to participants who were self-swabbing. Like other studies, majority of our participants found self-swabbing very easy with all women willing to provide subsequent self-collected swabs. ^33^^,^^34^ Notably, 71% preferred self-swabbing to healthcare provider-collected swabbing. Those who prefer healthcare provider collected samples could be due to greater confidence in provider sample collection and interaction with trained personnel as previously demonstrated. ^35^

Though our participant sample size was not sufficient to reach statistical significance, comparisons of self- to provider-collections suggest similar HPV detection which warrants further investigation in adequately powered studies. Overall, these findings support the implementation of affordable, dry-stored, swab-based HPV diagnostics in decentralized settings in accordance with other findings. ^14^^,^^36^ The ability to deploy a flexible point of care assay on existing infrastructure—without dependence on proprietary instruments—could significantly expand HPV screening capacity in low-resource settings to reduce the burden of cervical cancer globally, especially when paired with timely treatment.

## Ethical approval statement and consent

Study was approved by Salus IRB located at 2111 W. Braker Lane, Suite 100, Austin, Texas, 78758, www.salusirb.com. under Salus number 24219 i.e., Protocol number and title: 202403; Sensitive hrHPV Infection Early Low-cost Detection using Self-Collected Swabs (SHIELDS). Written Informed consent was obtained from all participants included in these studies.

## Data Availability

All data supporting these findings can be found within the manuscript and supplemental materials https://doi.org/10.6084/m9.figshare.32189805. ^37^ Data are available under the terms of the Creative Commons Attribution 4.0 International License (CC BY 4.0).
